# A multi-view contrastive learning for heterogeneous network embedding

**DOI:** 10.1038/s41598-023-33324-7

**Published:** 2023-04-25

**Authors:** Qi Li, Wenping Chen, Zhaoxi Fang, Changtian Ying, Chen Wang

**Affiliations:** 1 Shaoxing University, Shaoxing, 312000 Zhejiang China; 2 Chongqing University, Chongqing, 400030 China

**Keywords:** Applied mathematics, Computer science, Information technology

## Abstract

Graph contrastive learning has been developed to learn discriminative node representations on homogeneous graphs. However, it is not clear how to augment the heterogeneous graphs without substantially altering the underlying semantics or how to design appropriate pretext tasks to fully capture the rich semantics preserved in heterogeneous information networks (HINs). Moreover, early investigations demonstrate that contrastive learning suffer from sampling bias, whereas conventional debiasing techniques (e.g., hard negative mining) are empirically shown to be inadequate for graph contrastive learning. How to mitigate the sampling bias on heterogeneous graphs is another important yet neglected problem. To address the aforementioned challenges, we propose a novel multi-view heterogeneous graph contrastive learning framework in this paper. We use metapaths, each of which depicts a complementary element of HINs, as the augmentation to generate multiple subgraphs (i.e., multi-views), and propose a novel pretext task to maximize the coherence between each pair of metapath-induced views. Furthermore, we employ a positive sampling strategy to explicitly select hard positives by jointly considering semantics and structures preserved on each metapath view to alleviate the sampling bias. Extensive experiments demonstrate MCL consistently outperforms state-of-the-art baselines on five real-world benchmark datasets and even its supervised counterparts in some settings.

## Introduction

**Figure 1:**
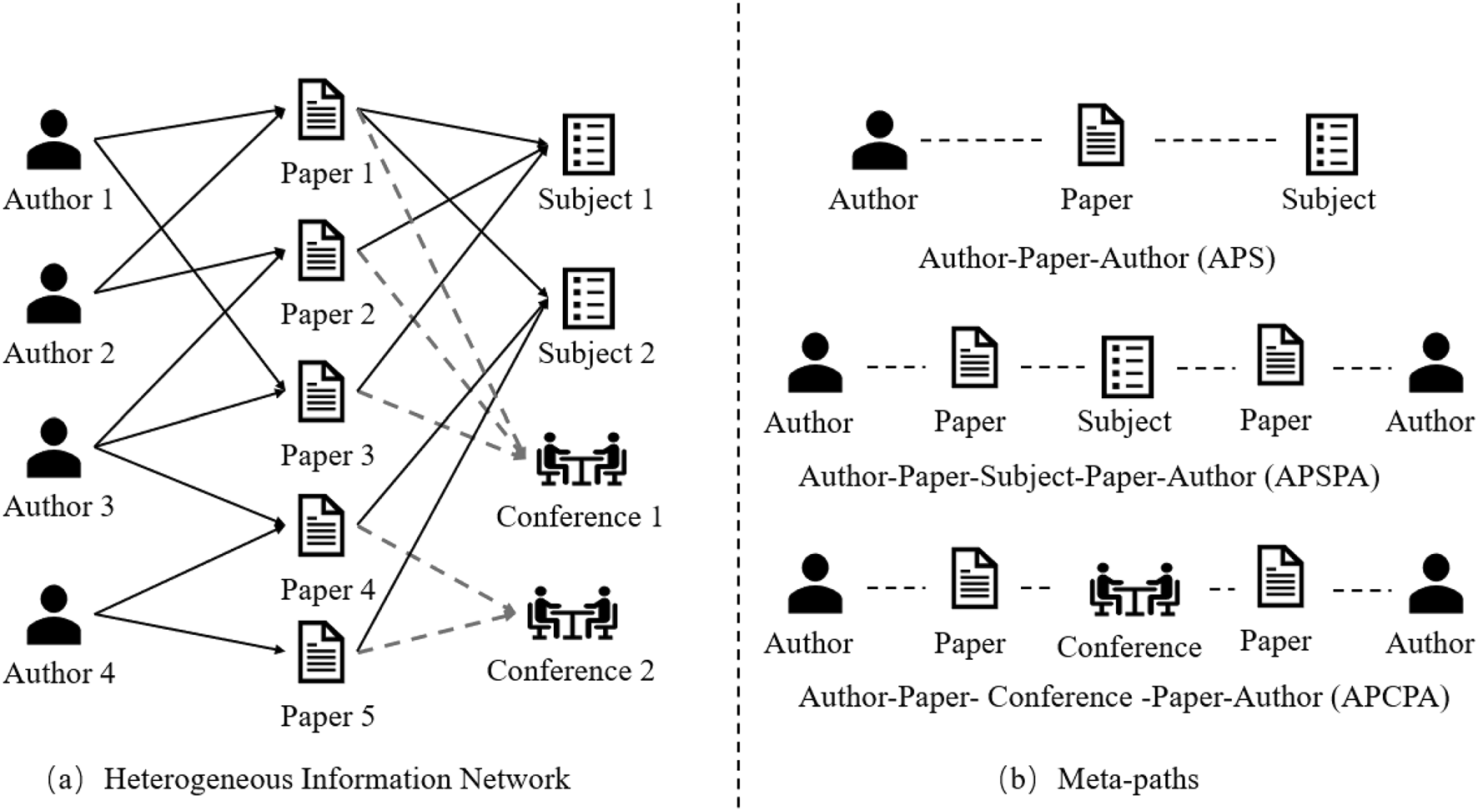
An example of heterogeneous information network.

Considering the capacity for modeling complex systems, Heterogeneous Information Networks (HINs) that preserves rich semantic information have become a powerful tool for analyzing real-world graphs^1–5^. As illustrated in Fig. 1, we present a concise example of a heterogeneous bibliography network with four types of nodes and three types of relations. Recently, Graph Neural Networks (GNNs)^6^ have emerged as a dominant technique in mining graph structure datasets, and its variant, Heterogeneous Graph Neural Networks (HGNNs)^4,7^, has occupied the mainstream of HIN analysis^8^. In general, HGNNs are trained in an end-to-end manner, which requires abundant, various, and dedicated-designed labels for different downstream tasks. However, in the majority of real world scenarios, it is highly expensive and difficult to collect labels^9^.

Contrastive learning (CL)^10–13^ that automatically generates supervised signals from data itself is a promising solution to learn representations in selfsupervised manner. By maximizing the confidence (mutual information)^14^ between positive pairs and minimizing the confidence between negative pairs, CL is capable to learn discriminative embeddings without explicit labels. Inspired by the success of CL in Computer Vision^11,12,15^, many Graph Contrastive Learning (GCL) methods have been proposed. For example, DGI^10^ exploits a contrast between graph patches (i.e., nodes) and graph summary, and GRACE^16^ maximizes the mutual information between the same node in two augmented views. Despite some works generalizing the key idea of CL to homogeneous graphs, there are still fundamental challenges that needs to be addressed in exploring the great potential of CL in heterogeneous graphs.

1. **How to design distinct views**? Data augmentation that creates corrupted views is shown to be an essential technique to improve the quality of representations^15,17^. In GCL, prevalent augmentations include edge dropping/adding, node dropping/adding, feature shuffling, and so forth. Although these methods excel in homogeneous graphs^16,18^, we argue that they materially change the latent semantics of HINs. Take a bibliographic network as an example (Fig. 1); if the link between Author 3 (A3) and Paper 4 (P4) is dropped, the closest path between Author 3 (A3) and Author 4 (A4) will be changed from 2-hop (A3-P4-A4) to 4-hop (A3-P4-S2-P5-A4). To prevent the knowledge altering brought by simple augmentations, we propose to leverage metapath, the composition of semantic relations, to augment datasets. By applying metapaths, we create multiple different yet complementary subgraphs, referred to as metapath views, without changing the underlying semantics while also capturing the high-order relationships on HINs.
2. **How to set proper pretext tasks**? The choice of pretext tasks (contrastive objective) determines the informativeness of representations in downstream tasks for GCL^19^. For HIN, the best choice of pretext tasks is still unclear and each work presents its own solution. For example, Park et al.^20^ proposes to use metapaths to learn a shared consensus vector as node representation, Wang et al.^21^ performs contrast between the aggregation of metapaths (view 1) and network schema (view 2), and Zhu et al.^22^ iteratively maximize the mutual information between a single metapath and the aggregation of them. Despite these approaches attempting to incorporate the universal knowledge across all metapaths, we argue that they actually assume metapaths are independent (different from the complementary nature), failing to capture the consistency between metapaths and thus leading to sub-optimality. To directly model the correlation between metapaths, we propose an intuitive yet unexplored pretext task that performs contrast between each pair of metapaths. To be specific, the contrast between two augmented views of a metapath (intra-metapath) aims to learn discriminative node representations and the contrast between two views generated on two sources (inter-metapath) ensures the alignment across metapaths.
3. **How to mitigate the sampling bias**? Sampling bias indicates that the negative samples randomly selected from the original datasets are potential to share the same class with the anchor (i.e., act as false negatives), which lead to a significant performance drop. Existing works^23–25^ select or synthesize true hard negatives to mitigate the issue. However, these methods are demonstrated to bring limited benefits or even impose adverse impacts on GCL^17^, because of the message passing^26^. To alleviate the false negative issue, we propose a positive sampling strategy that collaboratively considers topological and semantic information across metapaths to explicitly decide the positive counterparts for each anchor.

To summarize, we propose Multi-view Heterogeneous Graph Contrastive learning (MCL) to learn informative node representations for HINs. To the best of our knowledge, our work is the pioneer that treats metapaths as multi-views^15^ under the GCL framework and captures the complementarity between metapaths. In particular, we first apply metapath to create multiple views and leverage parameter-sharing GNNs to encode node representations. Then, we propose a novel contrastive objective that maximizes the mutual information between any pairs of metapath views (for both intra-metapath and inter-metapath) to explicitly model the complementarity among metapaths, which is neglected in other works. Specifically, we maximize the confidence between two metapaths at node-level and graph-level to acquire local and global knowledge. To further enhance the expressiveness, we propose a positive sampling strategy that directly picks hard positives for each node based on graph-specific topology and semantics to mitigate the sampling bias inherent in CL. We highlight the contributions as follows:

- We propose a heterogeneous graph contrastive learning framework, called MCL, which is the first attempt to treat metapaths in HIN as multiple views and leverages a novel pretext task to model the consistency between any pairs of metapath views at node- and graph-levels.
- We propose a positive sampling strategy, which selects the most similar nodes as positive counterparts for each anchor by considering semantics and topology across metapath views, to remedy the sampling bias.
- We conduct extensive experiments on five real-world datasets to evaluate the superiority of our model. Experimental results show MCL outperforms state-of-the-art self-supervised and even supervised baselines.

The remainder of this paper is organized as follows. In “Related work” section, work related to the heterogeneous network embedding technique is introduced; “Preliminary” section describes some preliminaries; “Multi-view heterogeneous graph contrastive learning” section describes the implementation of the multi-view contrastive learning for heterogeneous network embedding; “Multi-view heterogeneous graph contrastive learning” section provides the experimental results; finally, the research is summarized in “Experiments” section.

## Related work

GCL that marries the power of GNN and CL has emerged as an important paradigm to learn representations on graphs without annotations. As a pioneering work, DGI^10^ treats node embedding and graph summary as positive pairs and utilizes InfoMAX^14^ to optimize the objective. Following this line, MVGRL^27^ proposes to use graph diffusion as augmentations to generate multiple views and GraphCL^18^ further analyzes the role of augmentations in introducing prior knowledge. Inspired by instance discrimination^28^, GRACE^16^ and GCA^17^ proposes to leverage node-level objective in contrasting, preserving node-level discrimination. In addition, BGRL^29^ and its variants^26,30^ adopts the key idea of^31^ to perform contrast without negative samples via bootstrapping to save memory consumption.

Meanwhile, some studies have generalized the key idea of GCL on HINs. For instance, HDGI^32^ extends DGI to heterogeneous graphs and DMGI^20^ utilizes metapath encoder to train consensus vectors as node representations. CKD^33^ models the regional and global knowledge between each pair of metapaths, failing to capture node-level properties. CPT-HG^34^ applies relation-level and subgraph-level pretext tasks to pre-train HGNN on large-scale HINs, and HDMI^35^ introduces a triplet loss to further enhance generalization. However, these methods do not consider the sampling bias inherent in GCL, inevitably leading to sub-optimality. STENCIL^22^ and HeCo^21^ treat the aggregation of metapath-induced subgraphs as a novel view, and propose to apply metapath similarity to measure the hardness between nodes to synthesize hard negatives. However, they still fail to model the consistency and complementarity between metapath views. Our model remedies these deficiencies through (1) applying metapath as augmentation, (2) performing contrast between any pairs of metapaths at node-level and graph-level, (3) explicitly selecting positives for each node by considering topology and semantics.

## Preliminary

### Definition 1

Heterogeneous Information Network (HIN) refers as to the graph consisting of various types of nodes and edges, represented as $\mathcal {G}=\left\{ V,E,T,R \right\}$, where *V* and *E* are node set and edge set, respectively, and *T* and *R* denote node types and relation types, associated with a node mapping function $\psi :V\rightarrow T$ and a edge mapping function $\psi :E\rightarrow R$. Note that $\left| T \right| +\left| R \right| >2$.

### Definition 2

(*Metapath*) Meta-path $P_m$, $m\in M$ is the composition of relations in HINs, defined as $P_m=T_0\xrightarrow {R_0}T_1\xrightarrow {R_1}\ldots \xrightarrow {R_n}T_{n+1}$, where *M* is the set of meta-paths. *R* represents the type of relationship between nodes, and the subscripts 0,1,2,$\ldots$,*n* represent the types between different nodes. For example, $R_0$ represents the relationship between $T_0$ and $T_1$, and $R_1$ represents the relationship between $T_1$ and $T_2$. For example, we illustrate three meta-paths extracted from DBLP in Fig. 1, which describe co-author (APA), cosubject (APSPA), and co-conference (APCPA) relationships.

### Definition 3

(*Metapath-based neighbors*) Given a metapath $P_m$, meta-path-based neighbors $N_{v}^{P_m}$ is defined as a set of nodes connected to the target node through metapath $P_m$. For example, in Fig. 1, the meta-path-based neighbors of Author 1 via meta-path APA is Author 2.

## Multi-view heterogeneous graph contrastive learning

### Data augmentation

For HIN, collectively applying metapaths to construct multi-views is a natural way to supplement the dataset in opposition to simple augmentation techniques. The created multiviews are actually additive to each other because metapaths depict various facets of the same HIN. Given a set of metapaths $\left\{ P_0,P_1,\ldots ,P_{\left| M \right| } \right\}$, where $\left| M \right|$ is the number of metapaths. We extract multiple subgraphs (i.e., metapath views) $\left\{ g_0,g_1,\ldots ,g_{\left| M \right| } \right\}$ from the original graph to sustain the rich semantics preserved in HINs. For the subgraph $g_m$ generated through meta-path $P_m$, we construct the direct neighborhoods for each node *v* as its metapath-based neighbors $N_{v}^{P_m}$. Each metapath view is associated with a node feature matrix ${\textbf {X}}_{\varvec{m}}$ and an adjacent matrix ${\textbf {A}}_{\varvec{m}}$. We leverage parameter-sharing GNN encoders $f\left( \cdot \right)$ to learn node representation $\left\{ \textbf{H}_0,\ldots ,\textbf{H}_m,\ldots ,\textbf{H}_{\left| M \right| } \right\}$ from each metapath induced view, where $\textbf{H}_m=f\left( \textbf{X}_m,\textbf{A}_m \right)$. In practice, we leverage additional data augmentation (i.e., feature masking and edge dropping) with specific probabilities $p_f$ and $p_e$ to further corrupt metapath views to make the task more difficult.

### Contrastive objectives

To distill rich semantics in HINs, we propose a novel contrastive objective to maximize the correlation between any pair of metapath views. In particular, the contrastive objective is collaboratively performed in intra-metapath (i.e., contrast between two corrupted versions of a metapath view) and inter-metapath (i.e, contrast between two views from different metapaths). We argue that the intra-metapath contrast independently learns the augmentation-invariant latent for each metapath view and the inter-metapath contrast is to align the representations gained from various sources to acquire the complementarity inherent in metapaths. Thus, we thoroughly gain the underlying knowledge maintained in individual metapath views and explicitly model the dependencies between pairs of different metapath views. In addition, the pretext task between two views jointly learns from node and graph-level knowledge to enhance representativeness. Notice that in the node-level contrasting, we select hard positives via the proposed sampling strategy to mitigate the sampling bias.

#### Node–node contrast

Node-node contrast aims to learn discriminative node representations to boost node-level downstream tasks. Specifically, we perform contrast between the anchor and its positive counterparts in two views to maximize (resp. minimize) the confidence between similar (resp. unassociated) nodes:

1 $$\begin{aligned} \mathcal {L}_{local}^{\left( m,n \right) }\left( u,P_u \right) =-\log \frac{\sum _{v\in P_u}{\theta \left( h_{u}^{m},h_{v}^{n} \right) }}{\sum _{v\in P_u}{\theta \left( h_{u}^{m},h_{v}^{n} \right) }+\sum _{v\in \left( V\backslash P_u \right) }{\theta \left( h_{u}^{m},h_{v}^{n} \right) }+\sum _{v\in \left( V\backslash P_u \right) }{\theta \left( h_{u}^{m},h_{v}^{n} \right) }} \end{aligned}$$

where $h_{u}^{m}$ is the representation for node *u* in view *m*, $P_u$ denotes the selected positive samples for *u*. We use similarity function $\theta \left( h_{u}^{m},h_{v}^{n} \right) =e^{\varphi \left( \rho \left( h_{u}^{m} \right) ,\rho \left( h_{v}^{n} \right) \right) /\tau }$ to compute distance between node representations, where $\varphi \left( \cdot ,\cdot \right)$ measures the cosine distance between two vectors, $\rho \left( \cdot \right)$ denotes a nonlinear projector head that increases the expressiveness, and $\tau$ controls the data distribution. This objective function that pulls semantically similar nodes close and pushes dissimilar nodes away contributes to the discrimination of node representations.

#### Node–graph contrast

Different from node-node contrast that learns local semantics across multi-views, we also perform node-graph contrast as the auxiliary task to facilitate the representation learning by injecting metapath-specific knowledge. We define the following objective:

2 $$\begin{aligned} \mathcal {L}_{global}^{\left( m,n \right) }\left( u \right) =-\log \left( D\left( h_{u}^{m},s_m \right) \right) -\log \left( 1-D\left( h_{u}^{n},s_m \right) \right) \end{aligned}$$

where $s_m$ is the graph summary of metapath view $g_m$ calculated via the function *READOUT*($\cdot$) (mean pooling in the paper), and $D\left( h,s \right) =\omega \left( \rho \left( h \right) ,\rho \left( s \right) \right)$, where $\omega \left( \cdot ,\cdot \right)$ is a discriminator that consists of a bilinear layer $BiLinear\left( \cdot \right)$ and a sigmoid function $\sigma \left( \cdot \right)$. By imparting global knowledge brought by metapaths, we ensure the representations of nodes are more informative.

#### Overall objective

The overall objective to be maximized is defined as the aggregation of all pairs of metapaths, formally given by

3 $$\begin{aligned} J=\sum _{m\in M}{\sum _{n\in M}{\sum _{u\in V}{\mathcal {L}_{local}^{\left( m,n \right) }\left( u,P_u \right) }+\mathcal {L}_{global}^{\left( m,n \right) }}\left( u \right) } \end{aligned}$$

where *M* is the set of metapaths. After optimizing the contrastive objective, we perform late fusion $\eta \left( \cdot \right)$ (sum or concatenation) on node representations learned from multiple metapath views to get the unified node representations for downstream tasks.

4 $$\begin{aligned} h_u=\eta \left( \left\{ h_{u}^{m},m\in M \right\} \right) \end{aligned}$$

### Positive sampling strategy

**Figure 2:**
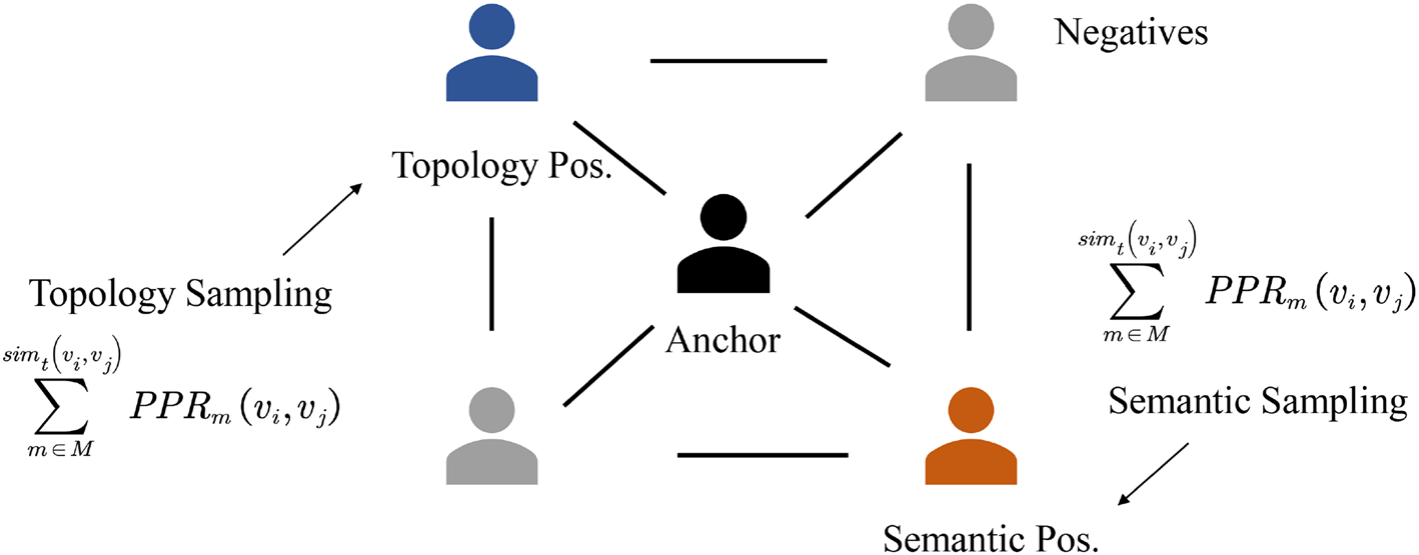
The positive sampling strategy. Personalized PageRank (PPR) is used to measure the topological similarity between nodes, and L2 distance is leveraged to compute the distance between nodes in semantic space to discover semantic associations.

Sampling bias is an important problem in CL since false negatives will generate adverse signals. However, existing debiasing techniques are theoretically and empirically verified to bring severer sampling bias on GCL because the message passing smooths the node representations. To overcome the deficiency, we propose to leverage two different yet reciprocal similarity measurements (i.e., topology and semantics) to define the distance between nodes, shown in Fig. 2, and explicitly select the most similar nodes as positive samples.

#### Topology positive sampling

To analyze the similarity between nodes based on topological structure, we propose to use diffusion kernel that assesses the global node importance to compute the distance between two arbitrary nodes. In practice, we apply Personalized PageRank (PPR) score to measure the node-level relationship for each metapath view.

5 $$\begin{aligned} \textbf{S}_m=\sum _{k=0}^{\infty }{\alpha \left( 1-\alpha \right) ^k\left( \textbf{A}_m\textbf{D}_m^{-1} \right) ^k} \end{aligned}$$

where $S_m\in \mathbb {R}^{\left| V \right| \times \left| V \right| }$, $\textbf{A}_m$, $\textbf{D}_m$ are diffusion matrix, adjacent matrix and diagonal degree matrix for metapath *m*, respectively, and $\alpha$ denotes teleport probability (default is 0.85). Formally, we define the *PPR* similarity between two nodes $v_i$ and $v_j$ under semantic view $g_m$ as the *i*-th row and *j*-th column in the diffusion matrix $PPR_m\left( v_i,v_j \right) =\textbf{S}_m\left[ i,j \right]$. The value in fact describes the stationary probability of starting from $v_i$ to reach $v_j$ via an infinite random walk in the metapath view *m*. Then, we aggregate the *PPR* scores computed on all metapath-induced views to determine the topological similarity.

6 $$\begin{aligned} sim_t\left( v_i,v_j \right) =\sum _{m\in M}{PPR_m\left( v_i,v_j \right) } \end{aligned}$$

and select the top-*k* similar nodes for each anchor as the topology positives $P^t$.

#### Semantic positive sampling

Apart from structural information, graph datasets also preserve rich semantics on the node itself. To measure the semantical similarity between nodes, we propose to utilize a simple metric (i.e., *l*2 distance) to compute the distance between attributes of nodes

7 $$\begin{aligned} sim_s\left( v_i,v_j \right) =-l2\left( x_i,x_j \right) \end{aligned}$$

where $x_i$ and $x_j$ denote attributes on nodes $v_i$ and $v_j$, respectively. The attributes for each node will not change across metapath views, thus we only need to process once to calculate the distance between pairs of nodes. Finally, we also select the top-*k* similar nodes for each anchor as the semantic positives $P^s$. At the time, we define the positive samples for node *u* across metapath views as

8 $$\begin{aligned} P_u=P_{u}^{t}\cup P_{u}^{s} \end{aligned}$$

Note that the positive sampling phase is performed in preprocessing, therefore, the module will not significantly increase the computational complexity.

## Experiments

### Datasets and baselines

To demonstrate the superiority of MCL over state -of-the-art algorithms, we conduct extensive experiments on five public benchmark datasets, including ACM, DBLP, IMDB, Aminer, and FreeBase. The statistics of five benchmark datasets as follows (Table 1).

**Table 1:** The statistics of five benchmark datasets.

Dataset	Nodes	Relations	Meta-paths
ACM	Author(A):7167	P-A:13407	PAP
	Paper(P):4025	P-S:4025	PSP
	Subject(S):60		
DBLP	Author(A):4057	P-A:19645	APA
	Paper(P):14528	P-C:14328	APCPA
	Subject(S):7723	P-S:85810	APSPA
	Conference(C):20		
IMDB	Actor(A):5257	M-A:12828	MAM
	Movie(M):4278	M-D:4278	MDM
	Director(D):2081		
AMiner	Paper(P):6564	P-A:18007	PAP
	Author(A):13329	P-R:58831	PRP
	Reference(R):35890		
FreeBase	Movie(M):3492	M-A:65341	MAM
	Actor(A):33401	M-D:3762	MDM
	Direct(D):2502	M-W:6414	MWM
	Writer(W):4459		

ACM^36^ is a bibliographic network which contains author, paper, and subject. According to the published conference, papers are labelled into three classes, i.e., data mining, database, and communication.

DBLP^8^ is extracted from a computer science bibliography website. Authors are divided into four groups based on research interests, including database, data mining, artificial intelligence, and information retrieval.

IMDB^8^ collects movies with actor and director information from online movie database. Movies are categorized according to genres, including action, comedy, and drama.

AMiner^21^ is another bibliographic graph where papers are labeled into 17 classes. We select a subset of the original graph with 4 types of papers. The initial node features are generated by DeepWalk.

FreeBase^21^ is a large knowledge network consisting of movie, actor, director, and writer. The movies are labeled into 3 genres. The initial node features are generated by DeepWalk.

We evaluate the performance of our model against various baselines from shallow graph representation learning algorithms, including DeepWalk^37^, Metapath2vec^38^, HIN2vec^39^, HERec^40^, to GCL methods (e.g., DGI^41^, GRACE^16^, DMGI^20^, STENCIL^22^, HeCo^21^) to supervised GNNs, like GCN^42^, GAT^10^, HAN^36^. Note that DMGI, STENCIL, HeCo are three GCL models dedicated for HIN.

### Evaluation protocol

We evaluate MCL on node classification and node clustering. For node classification, we use Micro-F1^40^ as the metric and follow the linear protocol that utilizes the learned graph encoder as a feature extractor to train an SVM classifier with 20% random samples as the training set. For node clustering, we apply K-means to generate clusters and utilize NMI as the metric. To mitigate the impact of initialized centroids, we perform 10 times clustering and report the average results. For all baselines, we run 10 times and present the average scores with standard deviations. For DGI, GRACE, GCN, GAT, we create homogeneous graphs based on metapaths and report the best results.

### Implementation details

We leverage a 2-layer GCN as the encoder for each metapath-induced view. The parameters are initialized via Xavier initialization^43^ and we apply Adam as the optimizer^44^. We perform grid search to tune the learning rate from 5*e*-4 to 5*e*-3, the value of temperature from 0.2 to 0.8, the corrupt rate from 0.1 to 0.7, the number of positives from 0 to 128. Moreover, we set early stop to 20 epochs, node dimension to 64, activation function to *ReLU*($\cap$) = *max*($\cap$,0), and use concatenation as fusion function in ACM and DBLP, and summation in other datasets. We present settings on baselines in the appendix.

### Quantitative results

We report the quantitative result of node classification and node clustering with standard deviations in Tables 2 and 3. From the tables, we observe that GCL methods generally perform better than shallow unsupervised baselines, since the instance discrimination applied on CL captures underlying semantics preserved in HINs but the graph reconstruction adopted in classical methods only considers the topological structure. Our models (MCL and MCL-P) consistently outperform state-of-the-art self-supervised graph learning methods across all datasets by a large margin on supervised classification and unsupervised clustering tasks, and even achieve competitive results compared to supervised baselines. Beyond that, the performance of MCL-P (positive sampling version) is commonly better than its vanilla version that performs contrast between the same node in different views, demonstrating the necessity of introducing correlated nodes as positives to mitigate the sampling bias. Compared with heterogeneous graph contrastive learning methods (DMGI, STENCIL, HeCo), our model always acquires higher scores in both classification and clustering. We argue that it is because (1) the intra-metapath contrast is performed between nodes on two corrupted views induced from the same metapath to learn the discriminative representations and (2) inter-metapath contrast captures the complementarity between metapaths instead of treating the aggregation of them as a single view under the independent assumption applied in mentioned baselines.

**Table 2:** Performance of node classification on five benchmark datasets.

Methods	ACM	DBLP	IMDB	Aminer	FreeBase
DeepWalk	81.78	88.09	56.36	84.93	69.63
MP2vec	79.82	87.67	50.78	84.14	69.66
HIN2vec	85.23	91.45	50.73	80.77	67.42
HERec	67.15	90.75	49.12	80.63	68.04
DGI	88.44	90.16	52.07	83.43	69.25
GRACE	87.64	91.28	54.80	83.43	69.25
DMGI	76.76	91.62	51.16	79.19	67.69
STENCIL	88.23	92.56	57.83	84.61	68.26
HeCo	88.97	92.24	52.12	85.22	69.02
**MCL**	91.02	93.29	60.75	86.63	71.41
**MCL-P**	**91.34**	**93.44**	**61.02**	**87.03**	**71.53**
GCN	89.87	92.04	58.42	85.42	69.13
GAT	88.84	92.51	54.78	84.37	70.42
HAN	89.53	93.27	54.78	85.91	70.98

Boldfaces denote the best performance among self-supervised and supervised methods, respectively. For our model, we use the suffix -P to indicate the positive sampling version.

**Table 3:** Performance of node clustering on five benchmark datasets.

Methods	ACM	DBLP	IMDB	Aminer	FreeBase
DeepWalk	41.15	20.13	5.97	30.17	14.56
MP2vec	37.74	73.77	2.71	26.52	14.93
HIN2vec	40.79	68.83	3.88	23.76	14.26
HERec	45.39	70.38	4.39	31.05	15.32
DGI	43.47	54.44	4.09	29.82	15.16
GRACE	46.51	67.98	1.58	24.12	16.23
DMGI	52.53	67.41	5.45	28.32	12.35
STENCIL	56.67	71.41	8.25	29.99	13.19
HeCo	56.93	70.03	7.41	30.61	12.07
**MCL**	**65.13**	**73.28**	**9.34**	**36.12**	**15.46**
**MCL-P**	**65.75**	**74.53**	**8.95**	**35.62**	**16.26**
GCN	58.14	77.71	8.59	37.82	15.77
GAT	62.22	72.06	8.04	36.81	15.44
HAN	60.98	78.21	6.83	35.37	16.38

Boldfaces denote the best performance among self-supervised and supervised methods, respectively. For our model, we use the suffix -P to indicate the positive sampling version.

### Ablation study

#### Pretext task

To verify the role of each component in the contrastive objective, we perform ablation studies, shown in Table 4, to compare the performance of multiple variants on node classification. From the table, we observe that (1) Node-node contrast provides better discrimination ability compared with node-graph contrast since the fine grained information (patches) is leveraged in learning representations. When the node-level and graph-level objectives are jointly optimized, the performance is significantly improved, showing the necessity of simultaneously modeling local-level and global-level dependencies. (2) The intra-metapath contrast is essential in promoting the learning procedure, reflected in the competitive performance obtained in the initialized variant (Intra- & Node) against state-of-the-art self-supervised baselines. (3) The variant with full components persistently achieves the best performance since the intra-metapath contrast captures the latent semantics of each metapath-induced view and the inter-metapath contrast aligns the consistency between metapaths. If one of them is removed, we cannot thoroughly model the relationship between metapaths, thus encountering model degradation.

**Table 4:** Ablation study of pretext tasks on node classification where the intra- and inter- are abbreviations of intra-metapath and inter-metapath contrasts, and local and global indicate node-node and node-graph contrasts, respectively.

Intra-	Inter-	Local	Global	ACM	DBLP	IMDB	Aminer	FreeBase
$\surd$	–	$\surd$	–	89.21	91.94	58.71	84.95	69.61
$\surd$	–	–	$\surd$	83.21	90.95	48.56	83.42	69.38
$\surd$	–	$\surd$	$\surd$	90.52	92.52	60.12	86.17	71.16
–	$\surd$	$\surd$	$\surd$	88.32	92.44	59.83	85.92	70.65
$\surd$	$\surd$	$\surd$	$\surd$	91.02	93.29	60.75	86.63	71.41

#### Positive sampling strategy

We also conduct experiments to evaluate the impact of positive sampling, presented in Table 5. We can find that the selected positives indeed improve the performance by implicitly defining hard negatives. In addition, the significance of these two positive sampling strategies depends on the choice of datasets, i.e, there is no obvious superiority between topology positives and semantic positives. However, when they are simultaneously leveraged, our model achieves the best scores. We argue the phenomenon demonstrates that the selected positives based on different strategies are distinct yet complementary.

**Table 5:** Ablation study of positive sampling strategy on node classification with four variants; $\surd$ denotes the specific type of positives are selected.

Topology Pos.	Senamtic Pos.	ACM	DBLP	IMDB	Aminer	FreeBase
–	–	91.02	93.29	60.75	86.63	71.41
$\surd$	–	91.17	93.34	59.92	86.88	71.43
–	$\surd$	90.88	93.35	58.28	86.91	71.45
$\surd$	$\surd$	91.34	93.44	61.02	87.03	71.53

### Hyperparameter analysis

#### Positive sampling thresholds

**Figure 3:**
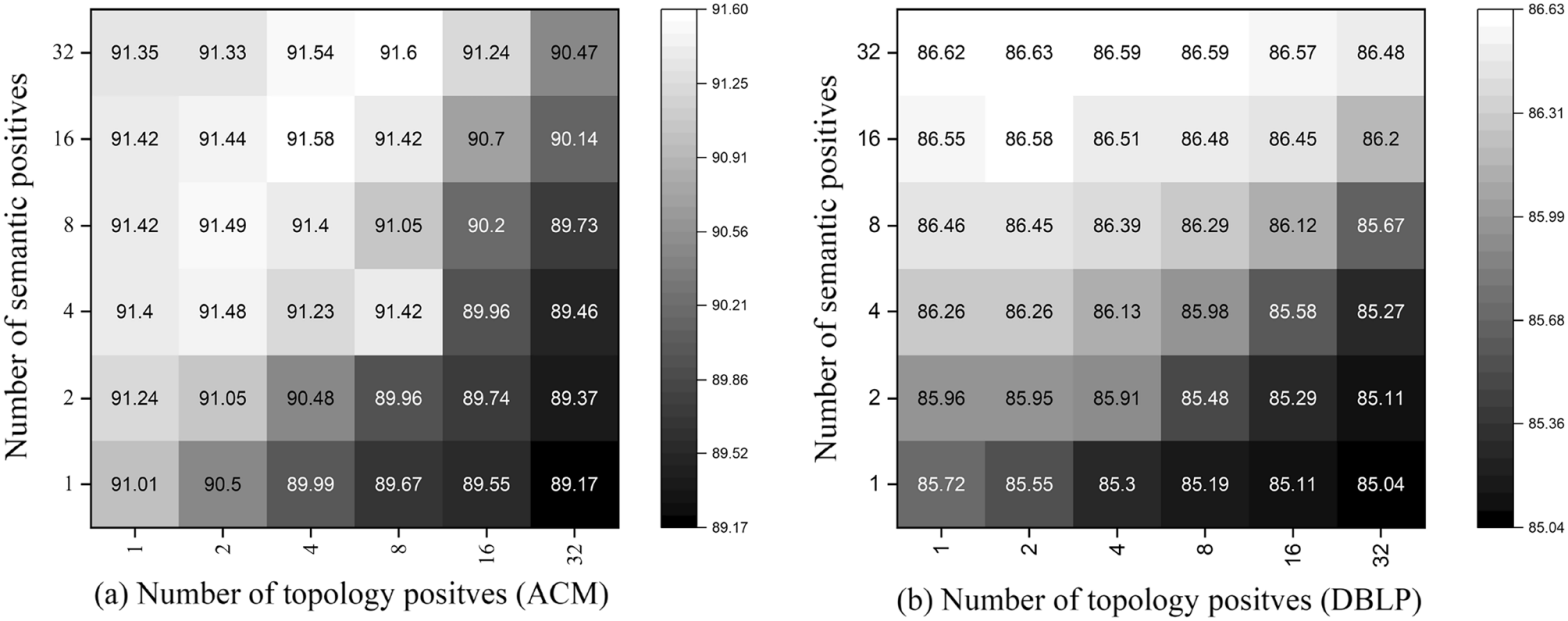
Hyperparameter sensitivity of positive sampling thresholds on node classification (ACM and DBLP).

**Figure 4:**
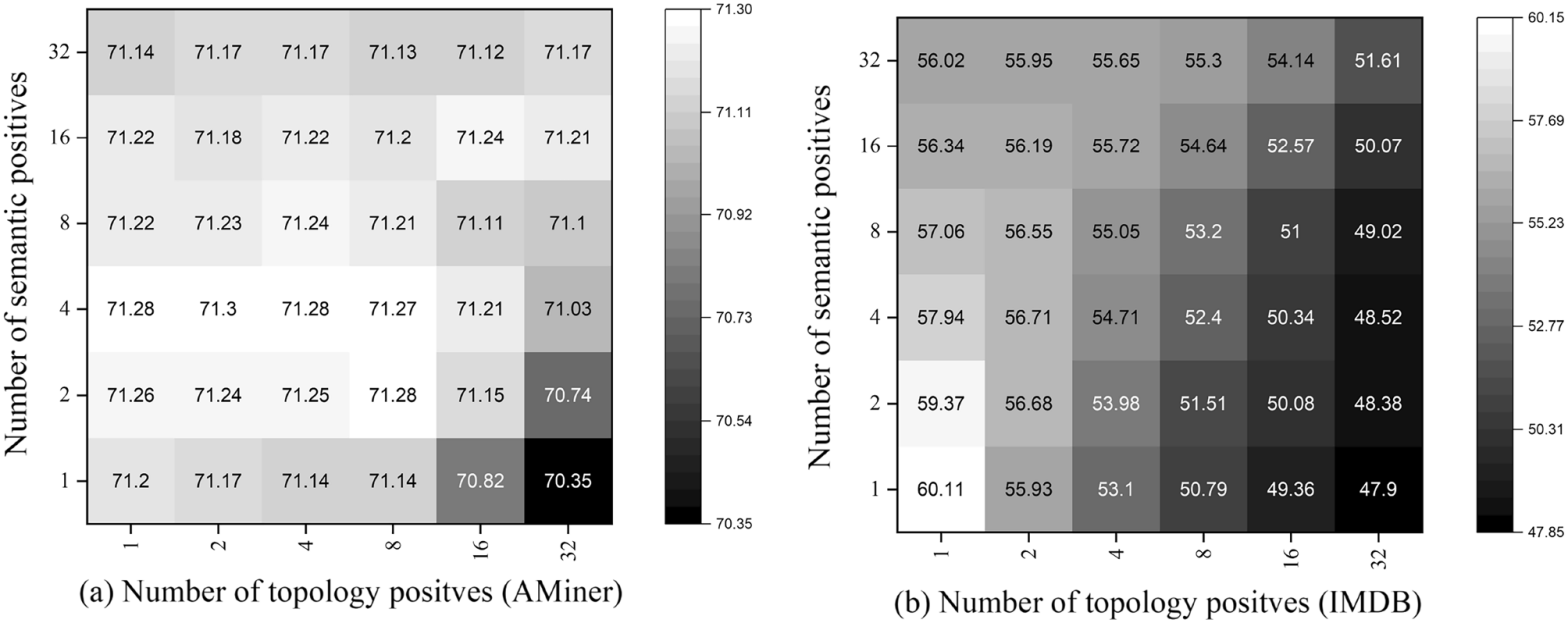
Hyperparameter sensitivity of positive sampling thresholds on node classification (AMiner and IMDB).

In the above section, we analyze the impact of positive sampling strategies in enhancing the quality of representations, here we delve into the positive sampling thresholds to provide a further examination.We illustrate multiple heatmaps that show the correlation between topology positives and semantic positives in Figs. 3 and 4. As we can see, the best performance is achieved with a large number of semantic positives and a small number of topology positives (ACM, DBLP, IMDB). When the number of topology positives is too large, the performance generally encounters a drop. We assume the phenomena derive from the inherent property of defined similarity functions. To be specific, the semantical similarity is independently measured on attributes of nodes in the representation space, whereas the topological similarity is calculated based on the adjacent matrix, which makes the function naturally biased to nodes with dense connections. Thus, when the number of topology positives is too large, there will contain too many noisy nodes. The Aminer does not follow the observation on the other datasets, whose best performance is achieved when the number of semantic and topology positives are both small. It is because the attributes on Aminer are generated by DeepWalk, a random walk-based algorithm that is biased to hub nodes in the learning procedure.

#### Augmentation probabilities

**Figure 5:**
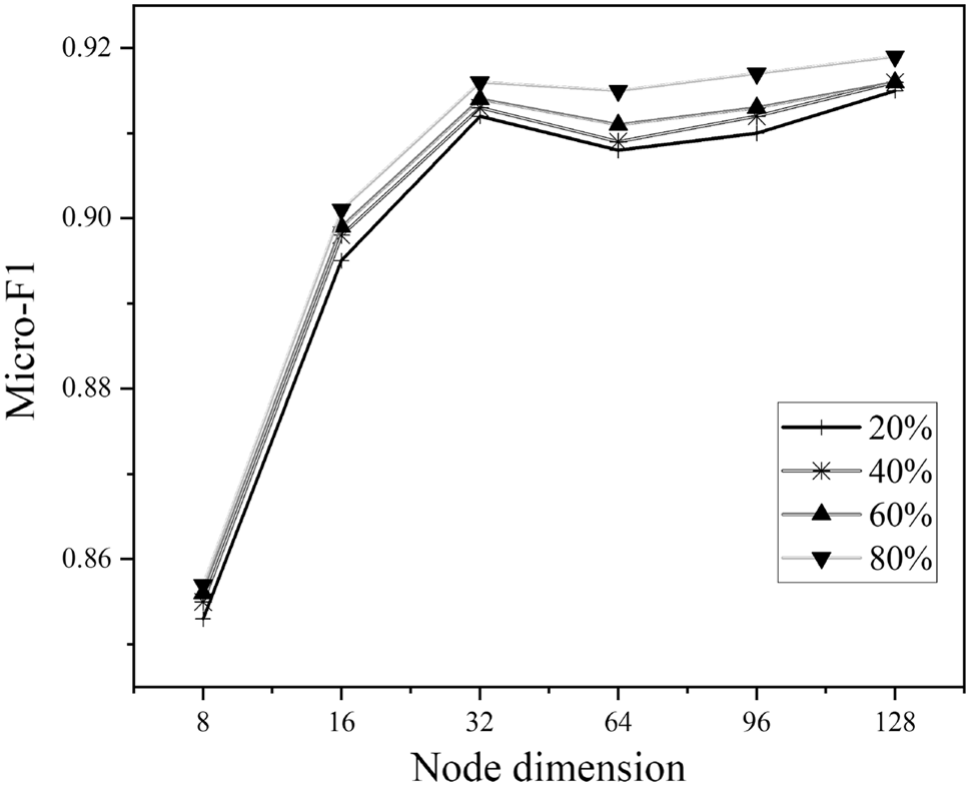
Hyperparameter sensitivity analysis on ACM.

**Figure 6:**
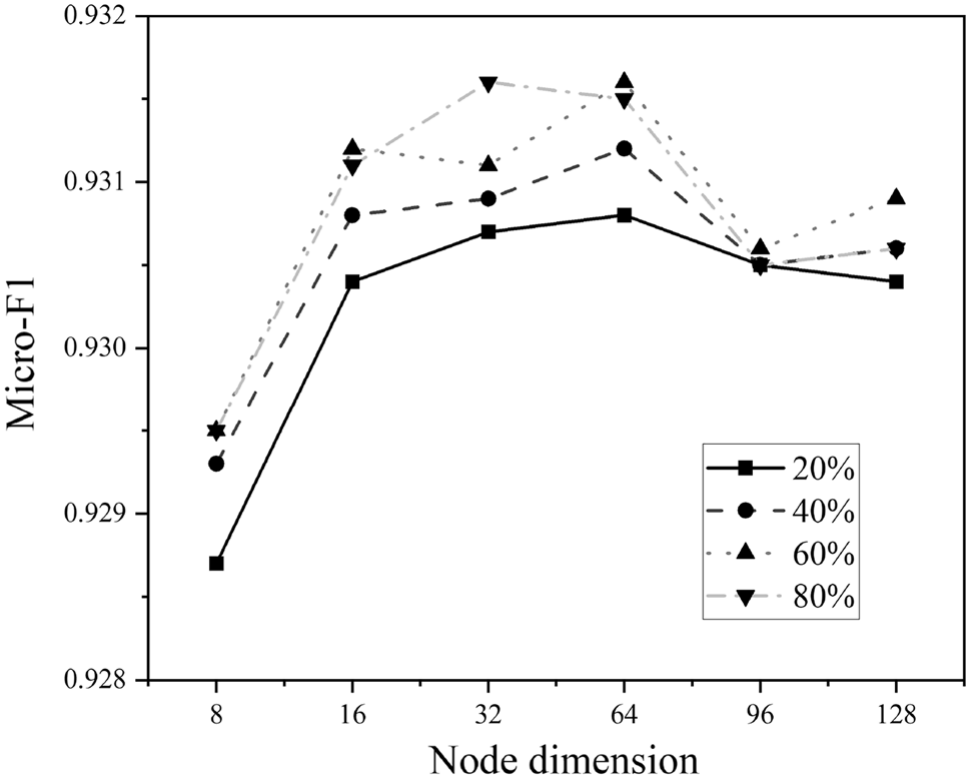
Hyperparameter sensitivity analysis on DBLP.

We evaluate the impact of node dimension on ACM and DBLP where the value varies in {8, 16, 32, 64, 96, 128}, illustrated in Figs. 5 and 6. As we can see, the best choice of node dimension is different across datasets. For ACM, the performance consistently increases with the increase of node dimension, whereas the curve of DBLP goes up first and then declined. However, they both perform well on a typical node embedding 64.

#### Temperature

**Figure 7:**
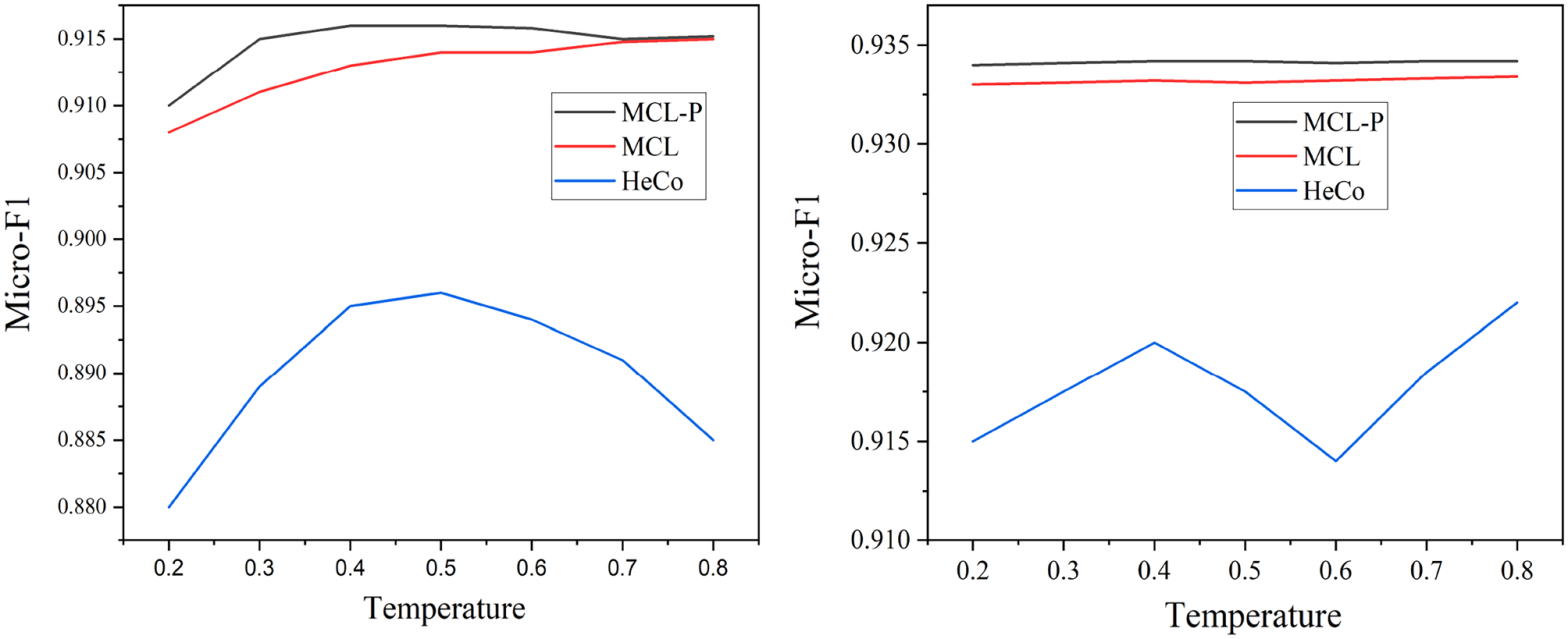
Hyperparameter sensitivity of temperature $\tau$ terms of Micro-F1.

The value of temperature determines the data distribution when measuring the distance between data points in contrasting. As illustrated in Fig. 7, we can see that our model is not sensitive to the temperature and have higher scores with lower variance against HeCo, showing its robustness. In addition, we observe that if the value of temperature is smaller, the gap between MCL-P and MCL will be larger. It is because the data distribution between positives and negatives will be smoother with the increase in temperature. The observation further proves the effectiveness of the proposed positive sampling strategy, especially with a small temperature.

### Visualization

**Figure 8:**
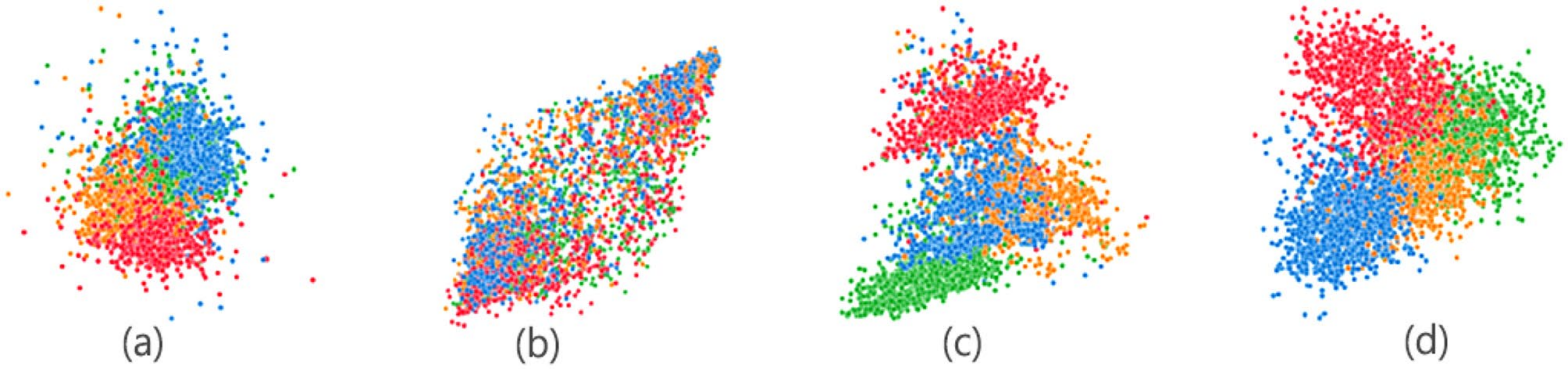
Visualization of node representations on DBLP (**a**) Metapath2vec (**b**) DMGI (**c**) HeCo (**d**) MCL.

To profoundly study the expressiveness of MCL, we visualize the learned node representations of DBLP through *t*-SNE. In Fig. 8, we visualize node representations obtained from four algorithms, including Metapath2vec, DMGI, HeCo, and MCL. As we can see, DMGI presents blurred boundaries between different classes, failing to learn discriminative low-dimensional node representations. For Metapath2vec and HeCo, despite some types of nodes being categorized clearly, there still exists a large proportion of overlapped data points that cannot be clearly identified. Our model separates nodes into different types, achieving the best performance.

## Conclusion

In this paper, we propose a multi-view heterogeneous graph contrastive learning framework named MCL. By treating metapaths as data augmentation, we create multi-views without impairing the underlying semantics in HINs. Then, we propose a novel objective that jointly performs intra-metapath and inter-metapath contrast to model the consistency between metapaths. Specifically, we iteratively utilize graph patches and graph summary to generate supervision signals to acquire local and global knowledge. To further enhance the quality of representations, we employ a positive sampling strategy that simultaneously considers node attributes and centrality to explicitly select positive samples to mitigate the sampling bias. Experimental results demonstrate the superiority of MCL across five real-world datasets on node classification and node clustering.

### Supplementary Information

Supplementary Information.

## Data Availability

The datasets used and/or analysed during the current study available from the corresponding author by request.

## Experimental settings

The architecture of GCN is defined as

9 $$\begin{aligned} \varvec{H}^{\left( l \right) }=GCN^{\left( l \right) }\left( \varvec{X},\varvec{A} \right) =\sigma \left( \varvec{\tilde{D}}^{-1/2}\varvec{\tilde{A}\tilde{D}}^{-1/2}\varvec{XW}^{\left( l \right) } \right) \end{aligned}$$

Where $\varvec{H}^{\left( l \right) }$ is encoded node representation at *l*-layer, $\varvec{\tilde{A}}=\varvec{A}+\varvec{I}$ is the adjacent matrix with self-loops, $\varvec{\tilde{D}}=\sum _i{\varvec{\tilde{A}}_i}$ is the degree matrix, $\sigma$ is a non-linear activation function, e.g., ReLU, and $\varvec{W}^{\left( l \right) }$ is the training weight for the *l*-th layer.

For random-walk-based and proximity-based methods, we set the walk length to 40, the context size to 10, walks per node to 20, the number of negative samples to 5. For homogeneous graph neural network, we test all metapaths and report the best performance. For fair comparison, we set node embedding to 64 for most baselines, the patience in early stopping to 20 epochs. To alleviate the instability derived from initialization, we repeat the experiments 10 times and report the average performance. For other settings, we follow the existing unsupervised heterogeneous graph learning.
