# Supplementary material for: A multi-view contrastive learning for heterogeneous network embedding
Source: Sci Rep. 2023 Apr 25;13:6732. doi: 10.1038/s41598-023-33324-7 (PMC10130187; doi:10.1038/s41598-023-33324-7)
Supplement: Supplementary file 1 — Supplementary Information. [file 41598_2023_33324_MOESM1_ESM.pdf]

1 Appendix

1.1 Datasets

To demonstrate the superiority of MCL over state -of-the-art algorithms, we conduct extensive experiments on five public benchmark datasets, including ACM, DBLP, IMDB, Aminer, and FreeBase. The statistics of five benchmark datasets as follows (Table 1).

ACM[1] is a bibliographic network which contains author, paper, and subject. According to the published conference, papers are labelled into three classes, i.e., data mining, database, and communication.

DBLP[2] is extracted from a computer science bibliography website. Authors are divided into four groups based on research interests, including database, data mining, artificial intelligence, and information retrieval.

IMDB[2] collects movies with actor and director information from online movie database. Movies are categorized according to genres, including action, comedy, and drama.

AMiner[3] is another bibliographic graph where papers are labeled into 17 classes. We select a subset of the original graph with 4 types of papers. The initial node features are generated by DeepWalk.

FreeBase[3] is a large knowledge network consisting of movie, actor, director, and writer. The movies are labeled into 3 genres. The initial node features are generated by DeepWalk.

Table 1. The statistics of five benchmark datasets.

Dataset	Nodes	Relations	Meta-paths
ACM	Author(A):7167<br>Paper(P):4025<br>Subject(S):60	P-A:13407<br>P-S:4025	PAP<br>PSP
DBLP	Author(A):4057<br>Paper(P):14528<br>Subject(S):7723<br>Conference(C):20	P-A:19645<br>P-C:14328<br>P-S:85810	APA<br>APCPA<br>APSPA
IMDB	Actor(A):5257<br>Movie(M):4278<br>Director(D):2081	M-A:12828<br>M-D:4278	MAM<br>MDM
AMiner	Paper(P):6564<br>Author(A):13329<br>Reference(R):35890	P-A:18007<br>P-R:58831	PAP<br>PRP
FreeBase	Movie(M):3492<br>Actor(A):33401<br>Direct(D):2502<br>Writer(W):4459	M-A:65341<br>M-D:3762<br>M-W:6414	MAM<br>MDM<br>MWM

1.2 Experimental Settings

The architecture of GCN is defined as

$$H^{(l)} = GCN^{(l)}(X,A) = \sigma\left(\tilde{D}^{-1/2}\tilde{A}\tilde{D}^{-1/2}XW^{(l)}\right) \tag{1}$$

Where $H^{(l)}$ is encoded node representation at l-layer, $\tilde{A} = A + I$ is the adjacent matrix with self-loops, $\tilde{D} = \sum_i \tilde{A}_i$ is the degree matrix, σ is a non-linear activation function, e.g., ReLU, and $W^{(l)}$ is the training weight for the l-th layer.

We present the specific hyperparameters for MCL in Table 2. For random-walk-based and proximity-based methods, we set the walk length to 40, the context size to 10, walks per node to 20, the number of negative samples to 5. For homogeneous graph neural network, we test all metapaths and report the best performance. For fair comparison, we set node embedding to 64 for most baselines, the patience in early stopping to 20 epochs. To alleviate the instability derived from initialization, we repeat the experiments 10 times and report the average performance. For other settings, we follow the existing unsupervised heterogeneous graph learning.

References

1. Wang, X. *et al.* Heterogeneous graph attention network. In *The world wide web conference*, 2022–2032 (2019).

2. Fu, X., Zhang, J., Meng, Z. & King, I. Magnn: Metapath aggregated graph neural network for heterogeneous graph embedding. In *Proceedings of The Web Conference 2020*, 2331–2341 (2020).

3. Wang, X., Liu, N., Han, H. & Shi, C. Self-supervised heterogeneous graph neural network with co-contrastive learning. In *Proceedings of the 27th ACM SIGKDD Conference on Knowledge Discovery & Data Mining*, 1726–1736 (2021).
